# The body asks and the mind judges: the episode of food craving, its triggers and nutritional treatment

**DOI:** 10.31744/einstein_journal/2022MD6705

**Published:** 2022-08-24

**Authors:** Jônatas de Oliveira

**Affiliations:** 1 Faculdade de Medicina Universidade de São Paulo São Paulo SP Brazil Faculdade de Medicina, Universidade de São Paulo, São Paulo, SP, Brazil.

**Keywords:** Feeding behavior, Feeding and eating disorders, Food preferences, Craving, Binge-eating disorder, Motivation

## Abstract

Food desires are defined as motivations that drive the search for and consumption of food. However, when domains of intensity and urgency are activated, these desires can become intense (*i.e.* food craving), being then characterized by episodes or cognitive events loaded with affectivity, in which food is associated with obtaining pleasure or relief, which is the only attentional focus. Specificity and urgency mark the differentiation between food desires and cravings. The process of elaboration with vivid images, the retention in working memory, the emergence of a negative affect state (awareness of the lack), and a committed attentional focus to seek food are characterized as stages of an episode of food craving. Individuals with eating disorders have the lowest levels of food craving when it comes to anorexia nervosa and the subsequent increase to bulimia nervosa and binge eating disorder. Some environmental and cultural triggers and internal factors of cognition and emotions play a crucial role in the emergence of food craving episodes. The external factors include positive/negative events, food environment, advertisements, cultural beliefs about food, specific locations, and food itself. The internal factors comprise dietary restriction, food reward, impulsivity/inflexibility, emotions, thoughts and feelings about food, hunger/satiety/appetite, and anxious/depressive symptoms. Treatment involves the association of flexibility, awareness, and questioning strategies about dietary practices based on three principles: unconditional permission to eat, eating more for physical than emotional reasons, and tuning in with the body’s signs of hunger and satiety (intuitive eating).

## INTRODUCTION

The punishment of Tantalus was about the tantalizing agony of desiring and yearning for something. To punish Tantalus, the gods made him stand neck-deep in water and, whenever he tried to slake his thirst, the water disappeared; and when he tried to reach for food, it was snatched from his grasp.^(1)^ Tantalizing means tormenting or torturing someone. This was also seen in volunteers in the famous Minnesota experiment, who, after being subjected to strict food restriction, reported presence and intensity of concerns and desires for food, as well as subsequent changes in their behavior.^(2)^

Elaborating how it would feel to eat a certain food and consciously suffering about it, in tantalizing agony, is one of the most current definitions for the intense desire for food known as food craving (FC). Historically, however, there has been much debate on the use of the term “craving”, with several definitions proposed. The construct was already studied and cited before there even was a clear definition, as seen in several publications that do not define FC, such as the article entitled “To depress the craving for food”,^(3)^ which includes studies in pregnant women^(4,5)^ and investigations of the pre-menstrual period.^(6)^ Nirenberg et al. pointed out the absence of a quantitative measurement for FC in studies of eating disorders (ED), which consider the involvement of cognitive elements in the determination of appetite.^(7)^ Weingarten et al. published one of the most cited articles with a definition of FC, and despite this data, throughout the entire review text, there is not a single sentence that defines the construct.^(8)^

Recently, Malika et al. interviewed women about their conceptualizations of FC, and one of them described it as follows: “It is as if you could taste it and smell it, while you think of it,”^(9)^ which goes against the definition proposed by Kavanagh et al., who defined food craving as “a cognitive event filled with affectivity, in which an object or activity associated with pleasure or relief breaks into attentional focus”,^(10)^ which can be applied to substances or food. Other authors define cravings as motivations that generate an impulse to search for a stimulus in the environment, which can be a source of pleasure or relief, and also refer to domains of great intensity.^(11)^ Currently, FC is understood as a desire for a specific food, which may be present through a cognitive event.

Nevertheless, there are different factors involved in the emergence of an initial thought or feeling that will trigger cravings. Thus, the purpose of this work is to describe the different characteristics of food craving, its intensity, its triggers and its relation with outcomes from the dysfunctional eating behavior, as well as to propose a nutritional treatment based on the principles of intuitive eating.

## THE FACETS OF DESIRE

Considering the motivational effect that desire has on appetite, as a driving force for the search for food, the intensity of the sense of desire can be measured by specific questions or scales (such as Visual Analogue Scales), in addition to questionnaires that evaluate the construct from a multidimensional perspective.^(12)^ On this basis, one can infer that desire is different from craving (as well as, in Portuguese, the terms *desejo versus fissura* would be used), and part of this differentiation is due to the intensity and urgency, but also to the different cognitive associations, and, when it comes to eating behavior, to the possibilities of “desire”, “desire for specific food” and “food craving” (Table 1).

**Table 1:** Desires; their intensities; their relation with physiological, affective and cognitive factors, and the probability of food consumption

Types of desire	Specificity	Factors		Cognitive aspect		Probability of consumption
	
		Physiological	Affective	Positive	Negative	
Desire	No	Maybe	Yes	Present	No	Low
Specific desire	Yes	Maybe	Yes	Present	No	Present and programmable
Food craving	Yes	Maybe	Intense	Present	Present and intense	High

Source: Oliveira J, Cordás TA. The body asks and the mind judges: food cravings in eating disorders. L´Encéphale. 2020;46(4):269-82;^(12)^ May J, Kavanagh DJ, Andrade J. The elaborated intrusion theory of desire: a 10-year retrospective and implications for addiction treatments. Addict Behav. 2015;44:29-34. Review.^(13)^ Boswell RG, Kober H. Food cue reactivity and craving predict eating and weight gain: a meta-analytic review. Obes Rev. 2016;17(2):159-77. Review.^(14)^

Individuals with ED show frequent episodes of FC and high levels of FC when reported or evaluated for behavioral tasks in comparison to others without ED. According to recent studies, the lowest FC levels are found in anorexia nervosa cases, followed by a gradual increase in bulimia nervosa, and the highest levels are present in binge eating disorder.^(12)^ A recent literature review of EDs found 12 possible triggers for FC, which were broken down into:

External: positive/negative events; food environment; advertisement; cultural beliefs about food; specific places and food itself.

Internal: dietary restriction; food reward; impulsivity/ inflexibility; emotions, thoughts and feelings about food; hunger/satiety/appetite and anxious/depressive symptoms.^(12)^

## THE FOOD CRAVING EPISODE

Contact with an external eating cue, such as viewing an advertisement, seeing an image, smelling food or hearing someone talk about food, can generate mental images of what the experience of eating would be like, and what the taste and smell of a food, or the pleasure of consuming, would feel like. If that food was consumed not so long ago, the probability of craving could be lower, however it would still exists. Said probability may be influenced by emotional and cognitive deprivation, *i.e*., despite the food having been recently consumed, and even in exaggeration, if there was no satisfaction, there is deprivation of it.^(10-12)^ Availability in the environment (external trigger 2), advertisements (external trigger 3) and specific places (external trigger 5) increase the possibility of consumption as a not-so-specific desire, but which will certainly be influenced by food preferences, previous experiences and memories which are very close to the moment of the final choice. If the desired food has not been consumed recently, the likelihood of desire is greater, because the possibility of the longing is greater,^(10)^ and the memory of how tasteful that food is can take over an important part of the cognition and flow of thoughts (specific desire, according to table 1), and it is possible to program consumption, according to the appropriate context.

In a third mode of manifestation of desire, when the individual is under the influence of internal triggers, such as dietary restriction (internal trigger 1) and food reward (internal trigger 2); impulsivity/inflexibility; emotions, thoughts and feelings about food; hunger/satiety/appetite and anxious/depressive symptoms,^(12)^ a sense of enchantment and anticipation of consumption can raise awareness of the lack and associate with deprivation and the possibilities - at this moment, already anxiogenic - of how nice it would be to consume that food. In ED, mental processes of self-control, dysfunctional beliefs about food, and momentary affection, occur in parallel.^(12)^ According to Kavanagh et al.^(10)^ thought captures the attention, and images of how to obtain food and how pleasant it would be to eat it invade the mind and accumulate in the working memory which, at the same time, generates discomfort due to lack, and increases the direction of attention focus in the unconsummated pleasure (tantalizing). This can occur in non-ED individuals who are under the influence of some triggers.^(2-7)^

Kavanagh et al.^(10)^ developed the elaborated intrusion theory of desire, arguing that psychological abstinence, environmental associations, cognitive associations, and negative affection are potential triggers for desires. Early awareness may be linked with a conditioned anticipated response, such as salivation, physiological excitation, and neural activity in regions such as the ventral striatum (food reward triggers and hunger/satiety/appetite triggers),^(12)^ which may cause a slight discomfort initially (as related to thoughts such as “I feel like eating something sweet”) and, therefore, it can activate representations in memory through automatic associative processes (in which, usually, the desire is specific). Thus, this stimulus increases the likelihood that a seemingly spontaneous thought will arise (intrusion into consciousness).^(5,13)^

## THE BODY ASKS AND THE MIND JUDGES: THE ROLE OF CONDITIONS FOR EATING (FOOD AND COGNITIVE RESTRICTION)

The elaborated intrusion theory of desire^(10)^ distinguishes associative processes, which can arise spontaneously, as when one sees the yellow ‘M’, and soon the mental image of a burger can emerge in consciousness while doing any other task, such as driving. This process depends on an association in memory, and the probability of this image appearing is much lower for those who have never frequented yellow ‘M’ stores. This example highlights how food advertising (external triggers 3 and 5: specific advertisements and places) and the creation of affective and appetizing memories can guarantee automatic and elaborative processes in the future. Non-specific desire can arise through the process of association, without any elaboration. In this case, one can consider the occurrence of the most frequent triggers, such as the food environment and advertising, but also the occurrence of an emotion commonly associated with the consumption of a food. In these cases, the individual is encouraged to question whether it is a desire or an emotion, and, if the desire is not specific, it may be a desire for relief from that emotion, *e.g*. “this is boredom and not desire for chocolate”.

When it comes to the elaboration process, consciousness and the search for a target must be present, with retention of information in the working memory (accumulation of images), also described by Kavanagh et al.,^(10)^ as a highly elaborated cognition about the target, more related to the specificity of the target (a specific food) and with a higher probability of consumption. Elaboration in the presence of other triggers, either internal or external, increases the likelihood of consumption, and the food itself is responsible for a small part of the components of the desire episode. In this case, more vulnerable individuals with a troubled relation with eating have a greater chance of elaboration and search for the target, in other words, the desire turns into craving. Great involvement of cognitive control for weight loss and body shape modification (*i.e.* cognitive restraint) ends up recruiting associated internal triggers with greater probability of consumption and lack of control,^(12)^ including dietary restriction, food reward, impulsivity/inflexibility and emotions, thoughts, and feelings about food.

Thoughts related to dietary control and distorted beliefs about eating are associated with the feeling of deprivation when one thinks about food consumption in an avoiding way, and thus the trigger of deprivation is reinforced whenever a food or cue in the environment evokes memory associations, anticipated salivation response, and other automatic associative processes.^(13)^ Thus, desire emerges and reinforces the cognitive restriction/diet mentality and the feeling of psychological deprivation, which may be increased by thoughts of self-control, which will try to suppress the emergence of intrusive thoughts, ultimately reinforcing them.^(15,16)^ The conflict between body responses (“the body asks”) and avoidances and beliefs (“the mind judges”) ends up increasing the intensity of desire when related to disordered eating behavior.

## NUTRITIONAL MANAGEMENT

Although FC is seen as a construct related with negative outcomes such as binge eating, emotional eating, snacking, and weight gain,^(12,14)^ understanding it seems like a better strategy than self-control.^(15-17)^ Hofmann et al.^(11)^ concluded, in a study on desires and self-control, that the frequency and recency of previous use of self-control strategies were associated with the failure to resist desires on the same day; that is, nutritional management poses the challenge of normalizing desire and understanding the triggers associated with increases in the intensity domains.^(12,18)^

**Figure 1:**
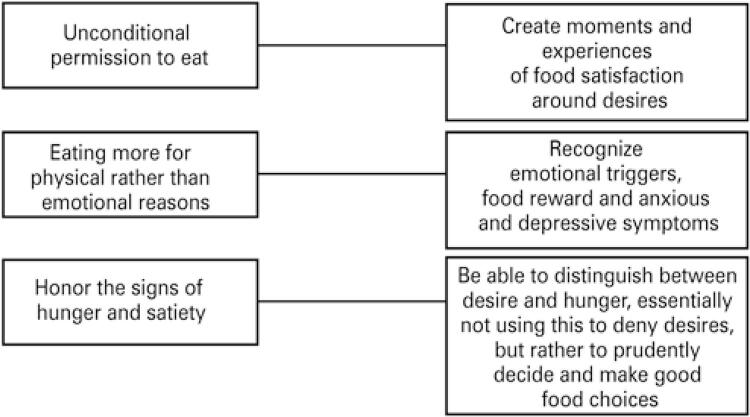
Association between the three principles of intuitive eating and eating experiences that involve desire for food

In the case of disordered eating, obesity or ED, one must understand that desiring food is part of a neural network and cognitive processes that reinforce a behavior that guarantees survival. This is a cultural aspect of eating that determines preferences, flavors and the pleasure of eating. Both physiological and cultural aspects are strongly denied and avoided in eating behavior targeted at body changes, as seen in ED,^(12)^ and nutritional management includes the recognition and naturalization of these processes. Many of the external triggers are counterintuitive factors for eating, *i.e.* conditions, rules, cultural beliefs or the influence of urgency, brought about by advertisements and the food environment.^(19)^ Modulating the environment with behavioral strategies is suggested (working on external triggers), but within this context, it is important to look at the modulation and challenging of beliefs about food, so that environmental modulation does not reinforce the notion that eating would be wrong or something that should be avoided. This is because the prohibition and suppression of thoughts do not contribute much to the treatment, and thus permission for eating is proposed in line with the needs of the body and its signs.^(20,21)^Therefore, the three principles of intuitive eating are associated with decreasing FC levels.^(20)^ The first, unconditional permission to eat, allows the individual to drop any rules for eating and the diet mentality, which recruits cognitive restriction as a process of self-control with food (Figure 1). On the other hand, unconditional permission to eat allows for the creation of food experiences that generate satisfaction of a desire, such as when specific desires are met. To this end, it is encouraged that a context of rejection of the diet mentality be created, in line with the second principle, which is eating more for physical than emotional reasons, which proposes a state of consciousness about potential triggers and affective states that drive eating for reasons other than hunger or genuine desire (Figure 1). The third principle of intuitive eating is to honor signs of hunger and satiety; in other words, when the presence and intensity of desire are recognized, it is possible to differentiate it from hunger, and thus one can decide, or at least understand, how food choices will be made when the desire occurs.

When one eats under an established condition or rule (counterintuitive process), the cultural aspect of food, associated with the pleasure of eating, is hardly satisfied, because although food consumption does occur, it is circumscribed in a rule, and this means that, when the condition or rule cannot be fulfilled, there is no permission to eat, because it depends on a condition/rule. The conditions established are present in popular discourse as ‘exercising gives you permission to eat’ or ‘eat on specific days’, such as the weekend, or ‘deserving’ food under intense emotional conditions.^(21)^

Food experiences that occur under rules and conditions do not lead to the same satisfaction, because food pleasure is attached to beliefs and thoughts of guilt (*e.g*., certain foods cannot be eaten on Mondays). Thus, cultural and sensory needs are not met, and soon there will be a feeling of physical and emotional deprivation.^(22)^ In cases of ED, there is great resistance in recognizing the social and affective role of food and that food consumption can occur beyond energy needs, such as in food cravings (whether selective or not). Eating episodes, such as binge eating and emotional eating, are preceded by episodes of FC in some cases.^(12,23)^ The recognition of triggers and the newly proposed greater dietary flexibility, such as that in intuitive eating, which includes the challenging of distorted beliefs about food, as well as the structuring of eating, contribute to the remission of binge eating and the decrease in FC frequency after treatment.^(19-22)^ Understanding the naturalness of desires and the consequence of controlling them is a matter infrequently discussed in the literature of ED, considering the negative connotation given to FCs due to them being predictors of binge eating, excessive eating and snacking. Future research should assess the relation between the intensity of desire and the number of triggers in self-reported FC or in association with behavioral tasks that lead to increased FC.

## CONCLUSION

Food craving is a temporal episode marked by affectivity, elaboration and anticipation of relief coming from a specific type of food, which is affected by external and internal triggers. The idea that some of these desired foods can lead to addictive eating behavior also prevents the naturalization of desire and increasingly demand cognitive restriction and self-control resources. Understanding the importance of trigger foods and that they can set off food cravings will shed more light on potential therapeutic measures. The intuitive eating approach has been shown effective when combined with challenging of dysfunctional beliefs and thoughts about food.
